# Identification of a RAD52 Inhibitor Inducing Synthetic Lethality in BRCA2-Deficient Cancer Cells

**DOI:** 10.3389/fphar.2021.637825

**Published:** 2021-04-29

**Authors:** Qianye Yang, Yu Li, Rong Sun, Jian Li

**Affiliations:** ^1^ Institute of Cancer Biology and Drug Discovery, Chengdu University, Chengdu, China; ^2^ Sichuan Industrial Institute of Antibiotics, Chengdu University, Chengdu, China; ^3^ West China School of Pharmacy, Sichuan University, Chengdu, China; ^4^ Basic medical research center, School of medicine, Nantong University, Nantong, China; ^5^ School of Medicine, Chengdu University, Chengdu, China

**Keywords:** Rad52, synthetic lethality, BRCA2 deficiency, molecular dynamics, self-association

## Abstract

The breast cancer susceptibility gene 1/2 (BRCA1/2) is frequently mutated in many malignant tumors, such as breast cancer and ovarian cancer. Studies have demonstrated that inhibition of RAD52 gene function in BRCA2-deficient cancer causes synthetic lethality, suggesting a potential application of RAD52 in cancer-targeted therapy. In this study, we have performed a virtual screening by targeting the self-association domain (residues 85–159) of RAD52 with a library of 66,608 compounds and found one compound, C791-0064, that specifically inhibited the proliferation of BRCA2-deficient cancer cells. Our biochemical and cell-based experimental data suggested that C791-0064 specifically bound to RAD52 and disrupted the single-strand annealing activity of RAD52. Taken together, C791-0064 is a promising leading compound worthy of further exploitation in the context of BRCA-deficient targeted cancer therapy.

## Introduction

Breast cancer is one of the most commonly diagnosed types of cancer in women throughout the world, contributing to about 30% of newly diagnosed cases and 14% of cancer-related deaths in women (Siegel et al., 2018). Breast cancer susceptibility gene 1/2 (BRCA1/2), one of the most comprehensively studied familial breast cancer hereditary factors, is frequently mutated in not only breast cancer (Tereschenko et al., 2002; Calderón-Garcidueñas et al., 2005) but also in other types of cancers, including prostate, ovarian, pancreatic cancer, and so on (Dutil et al., 2012; Kimberly et al., 2013), contributing to the pathogenicity of these malignancies (Kuchenbaecker et al., 2017).

Synthetic lethality describes the phenotype that only concomitant disruption of two genes will cause cell death, each of which is nonlethal alone (Hartwell et al., 1997), which provides an advantage to the noncancer cells without the driver mutation, such as those mutations found in BRCA1/2. The success of PARP inhibitors in treating BRCA1/2-mutated tumors has demonstrated the efficacy of cancer-targeted therapy exploiting synthetic lethality (Lord et al., 2015). The synthetic lethality between RAD52 and BRCA1/2 has long been established (Feng et al., 2011; Lok et al., 2013), identifying RAD52 as a potential therapeutic target against cancers with BRCA1/2 gene mutations. RAD52 has been shown to be involved in several aspects of DNA repair in mammalian cells (Nogueira et al., 2019). Structurally, Stasiak et al. (2000) observed that the full-length human RAD52 protein forms heptameric rings. Lloyd and colleagues found that the C-terminus 218–418 residues are responsible for the formation of higher order complexes by RAD52 rings (Ranatunga et al., 2001), while the crystal structures of the truncated RAD52 (residues 1–209) suggest an undecamer ring structure with a positively charged channel around its outer surface that likely interacts with ssDNA (Kagawa et al., 2002; Singleton et al., 2002). Biochemically, a series of studies have demonstrated that the RAD52 ring structure is critical to its activity in promoting annealing complementary DNA strands during different repair pathways (Grimme et al., 2010; Hanamshet et al., 2016). Disruption of the RAD52 ring structure, that is, abolishing RAD52 function in DNA repair, will lead to specific killing of the cell with BRCA deficiency.

A couple of studies have investigated the effect of treating BRCA-deficient cancer cells with RAD52 inhibitors discovered through various strategies (Chandramouly et al., 2015; Hengel et al., 2016; Huang et al., 2016; Sullivan et al., 2016; Jian et al., 2018). Here, we exploited a series of computational and biochemical assays to discover and characterize specific RAD52 inhibitors inducing synthetic lethality in BRCA2-mutated cancer cells. Among the 66,608 drug-like compounds screened, we identified one compound, C791-0064, that disrupted the self-association of RAD52 protein and inhibited the proliferation of BRCA2-deficient cells more potently than wild-type cells. C791-0064 could serve as a promising leading compound in future studies for targeted cancer therapy in the context of BRCA2 deficiency.

## Materials and Methods

### Virtual Screening and Molecular Dynamics.

Discovery of putative RAD52 inhibitors by virtual screening and molecular dynamics analysis of the RAD52-inhibitor complexes were performed as described in our previous work with optimized parameters for the current study (Jian et al., 2018). In brief, PDB entry 5JRB of RAD52 and ChemDiv PPI_1127222 library were used for virtual screening by a procedure of two-round docking. The SwissADME online tool was then used to predict the ADME property of docking-identified 28 putative inhibitors (Daina et al., 2017), followed by 27.5-ns MD simulations with GROMACS package (version 4.6.7) to analyze the RAD52-inhibitor interaction characteristics (Pronk et al., 2013)

### Chemicals, Protein, and Deoxyribonucleic Acid

Putative RAD52 inhibitors, C791-0064, E859-1790, F345-0611, G672-0331, and G889-2311, were obtained from ChemDiv (San Diego, CA, United States). RAD52 protein was expressed as a 6-histidine–tagged recombinant protein from *E. coli* and purified to homogeneity using Ni-affinity and heparin chromatography as described (Sugiyama and Kantake, 2009). The sequence of the 50-nucleotide long ssDNA is 5′- TAA​ATG​CCA​ATG​CTG​CTG​ATA​CGT​ACT​CGG​ACT​GAT​TCG​GAA​CTG​TAA​CG -3′, synthesized by BGI (Shenzhen China).

### Cell Lines, shRNA, and siRNA Knockdown

The BxPC3 cell line was obtained from the National Collection of Authenticated Cell Cultures of China, and the Capan-1 cell line was obtained from the American Type Culture Collection. BxPC3 cells were cultivated with RPMI 1640 containing 10% FBS and 1% penicillin–streptomycin (Gibco, Thermo Fisher Scientific, Waltham, MA, United States); Capan-1 cell cultivation media contained 20% FBS and 1% antibiotics. BRCA2 knockdown–stable cell lines were generated by lentivirus-mediated shRNA knockdown. The shRNA sequences targeting BRCA2 (sh-BRCA2-UTR: 5′-CGC​TTA​ACC​TTT​CCA​GTT​TAT-3′, sh-BRCA2-CDS: 5′-AGCTTA CCTTGAGGGTTATTT-3′) were cloned into lentiviral vector pLKO.1. Lentiviruses were generated by co-transfecting these plasmids into 293T cells with psPAX2 and pMD2.G using Lipofectamine 3000. BxPC3 cells were infected and screened with 2 μg of puromycin for 10 days, and BRCA2 knockdown was confirmed by Western blot analysis. siRNA transfection was performed according to the manufacture’s instruction of Lipofectamine 3000. The siRNA sequence targeting RAD52 was 5′-GGA GUG ACU CAA GAA UUA ATT-3′.

### Cell Viability Assay

Cells were plated in a 96-well plate for 2000 cells/well. Twenty-four hours after seeding, the cells were exposed to indicated concentrations of compounds. After 72 h of treatment, cell viability was analyzed with a CCK8 assay kit (7sea Biotechnology, Shanghai, China).

### Western Blotting

Western blots were performed using a standard procedure. In brief, protein electrophoreses were carried out on 10% SDS-PAGE gels, followed by PVDF membrane transferring, 10% nonfat milk in TBST blocking, and probing with antibodies against the protein of interest. An enhanced chemiluminescence detection kit (Pierce, Thermo Fisher Scientific, Waltham, MA, United States) was used for signal detection. The antibodies used in the study included anti-BRCA2 and anti-γ-H2AX (Abcam, Cambridge, MA, United States), anti-Bax and anti-Bcl2 (Cell Signaling Technology, Beverly, MA, United States), and anti-Histag and anti-actin (Proteintech, Wuhan, China).

### Electrophoretic Mobility Shift Assay

Human 6His-RAD52 protein (final concentration 1 μM) and C791-0064 (final concentration 100 μM) were added to a 25 μL reaction, containing 25 mM HEPES pH 7.5, 1 mM DTT, 10 mM MgCl_2_, 150 mM NaCl, and 0.1 mg/ml BSA. The reaction mixture was incubated at 37 °C for 3 min, followed by the addition of 400 μM of ssDNA oligo. The mixtures were incubated at 37 °C for another 3 min, mixed with glutaraldehyde to a final concentration of 0.1%, and further incubated for 3 min at 37 °C. The reaction was stopped by chilling on ice and analyzed by a 4–20% gradient SDS-PAGE gel (Sangon Biotech, Shanghai, China), detected by Western blot using anti-Histag antibody (Proteintech, Wuhan, China).

### Clonogenic Assay

Cells for colony formation were seeded in 6-well plates at 500 cells/well and treated with 40 μM of C791-0064 for 48 h. After that, the culture medium was replenished with fresh medium every 3 days. On the 14th day after seeding, the cells were fixed and then stained with 0.5% crystal violet.

### Single-Strand Annealing Assay

The single-strand annealing assay was adopted from the method used by Lloyd et al. (2002). In brief, purified RAD52 0.5 μM was preincubated with an increasing amount of C791-0064 (0, 20, 40, 60, 80, and 100 μM), then added to the p32 5′-end-labeled 54-base oligonucleotide (10 nM), followed by addition of a 105-base oligonucleotide (10 nM) with 54 complementary bases. Reaction products were resolved on 2% agarose gel and analyzed by Molecular Imager FX and QuantityOne software (Bio-Rad). The sequence of the 54-base oligonucleotide was 5’-GGC GGA GGC CAG AAG GTG TGC TAC ATT GCG GCT GCT CGG GTA ATT AAT CTG GCC-3’. The sequence of the 105-base oligonucleotide was 5’-GGC CAG ATT AAT TAC CCG AGC AGC CGC AAT GTA GCA CAC CTT CTG GCC TCC GCC GAT ATC GAC AAC CTG CTG TGC TCC CAG GAT ACG GGC GAG TTA GCT TGA ACG-3’.

### Immunofluorescence Staining

Cells were first plated on glass slide cover slips. At about 80% confluency, the cells were then treated with 40 μM of C791-0064 for another 24 h, fixed with formaldehyde, permeabilized with 0.5% Triton X-100, blocked with 2% bovine serum albumin, and stained with anti-γ-H2AX antibody, followed by a secondary antibody. 4’-6-diamidino-2-phenylindole (DAPI) was used for the counterstaining of the nuclei.

### Microscale Thermophoresis

Purified RAD52 proteins were labeled with fluorescence dye using the Monolith Protein Labeling Kit (NanoTemper Technologies, München, Germany). The assay buffer contained 20 mM Tris, pH 7.4, 0.3 M NaCl, 5% glycerol, 3% DMSO, and 0.05% Tween-20. The samples were analyzed with Monolith NT.115 standard glass capillaries after 30 min of incubation. The concentration of the labeled RAD52 was 1 μM, while that of C791-0064 was serially diluted.

### Statistical Analysis

Statistical analysis was performed based on the results from three independent replicates. GraphPad Prism 5.0 software was used for analysis, and the results were expressed as mean ± SD. Student’s t test was exploited to evaluate the differences between groups. P-values <0.05 were considered to be statistically significant.

## Results

### Identification of Putative RAD52 Inhibitors by Virtual Screening

**FIGURE 1:**
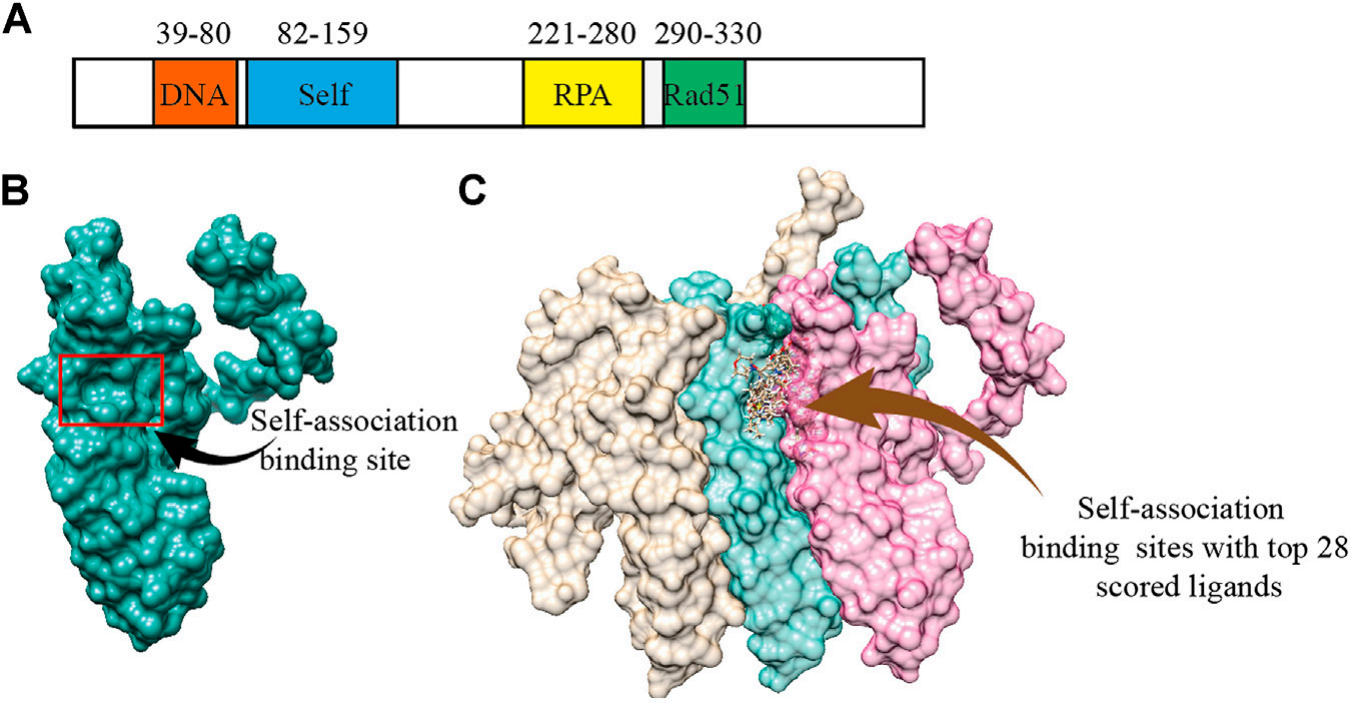
Human RAD52 self-association binding sites. **(A)** Functional domains of RAD52 protein. **(B)** Three-dimensional structure of RAD52 protein; the red box is the groove structure defined as the potential RAD52 self-association site. **(C)** Top 28 small molecules with the highest docking score bound to the self-association site of RAD52. The three colored structures represent three RAD52 monomers.

According to the previous biochemical study, we selected a groove surface in the self-association domain (ALA85-GLY159) of RAD52 as the docking site which has been shown to be critical for forming the functional RAD52 heptamer (Figures 1A,B) (Baoyuan et al., 2004). We screened a total of 66,608 compounds. The top 28 scored compounds that were successfully docked into the self-association domain of RAD52 are shown in Figure 1C. These compounds were then grouped into 12 groups according to structural similarity and evaluated for their AMDET properties (Supplementary Tables S1 and S2). Based on their drug-like properties, C791-0064, E859-1790, G889-2311, F345-0611, and G672-0331 were chosen for further analysis (Table 1).

**TABLE 1:** Structures and docking scores of representative putative RAD52 inhibitors.

Compounds	Structure	Grid score (kcal/mol)	Amber score (kcal/mol)
C791-0064	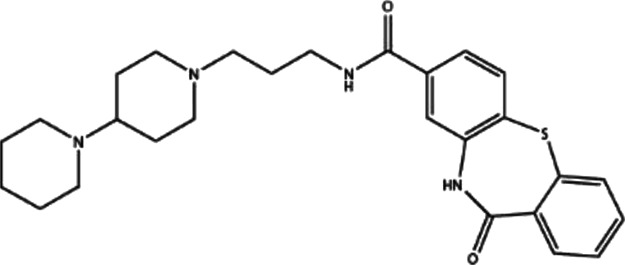	−41.37	−38.36
E859-1790	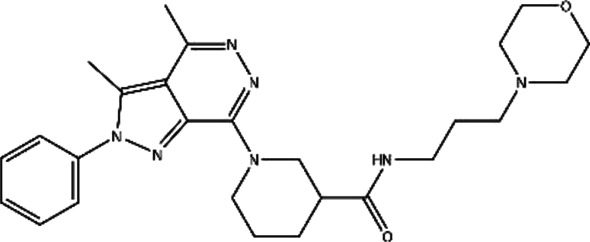	−45.35	−35.58
G889-2311	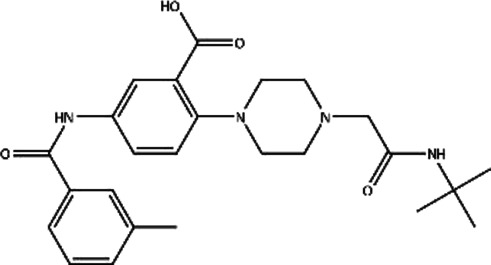	−38.49	−35.86
F345-0611	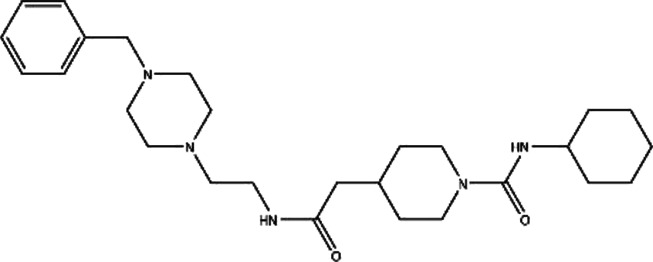	−41.92	−39.91
G672-0331	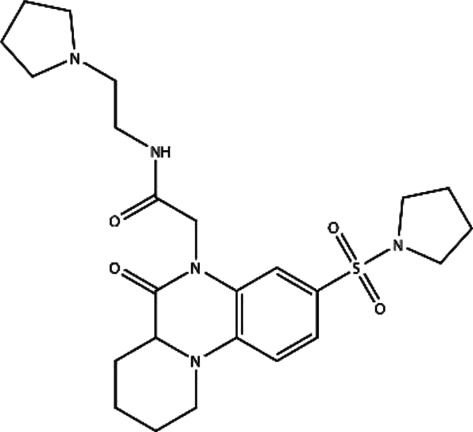	−38.36	−35.13

Among the five compounds, E859-1790 gave the lowest grid score (−45.35 kcal mol-1) in the first round of evaluation. After the amber scoring, the lowest score was assigned to F345-0661 (−39.91 kcal mol-1), which also had the second lowest grid score in the first round. Because the amber score exploits the “induced-fit” strategy, it simulates the receptor-ligand affinity more accurately. Therefore, F345-0611 was considered to be the molecule with the highest affinity to the RAD52 protein based on our grid and amber scoring analysis.

### Hydrogen Bonding and Hydrophobic Interaction Between RAD52 and Putative Inhibitors.

The five putative RAD52 inhibitors were able to be docked into the cleft responsible for the RAD52 self-association (Supplementary Figure S1). F345-0611 and G889-2311 were found to form only one hydrogen bond with the RAD52 amino acid residue. C791-0064, E859-1790, and G672-0331 were predicted to form two such hydrogen bonds. Arg112, His86, and Ser67 of RAD52 can form hydrogen bonds with the ligands inside the self-association groove, while Tyr81 and Asn76 can form hydrogen bonds with the ligand outside the groove.

Amino acid residues critical for RAD52 self-association were plotted with LigPlot + to investigate the RAD52–ligand interaction mechanism (Supplementary Figure S2). As can be seen from Supplementary Figure S2, in addition to the hydrogen bonding interaction, the amino acid residues, including Phe26, Ile72, Asn76, Tyr81, Asn82, Trp84, Ala85, His86, Ser87, and Gln114, hydrophobically interacted with the candidate RAD52 inhibitors.

### Molecular Dynamics Simulation of RAD52–Ligand Interaction

**FIGURE 2:**
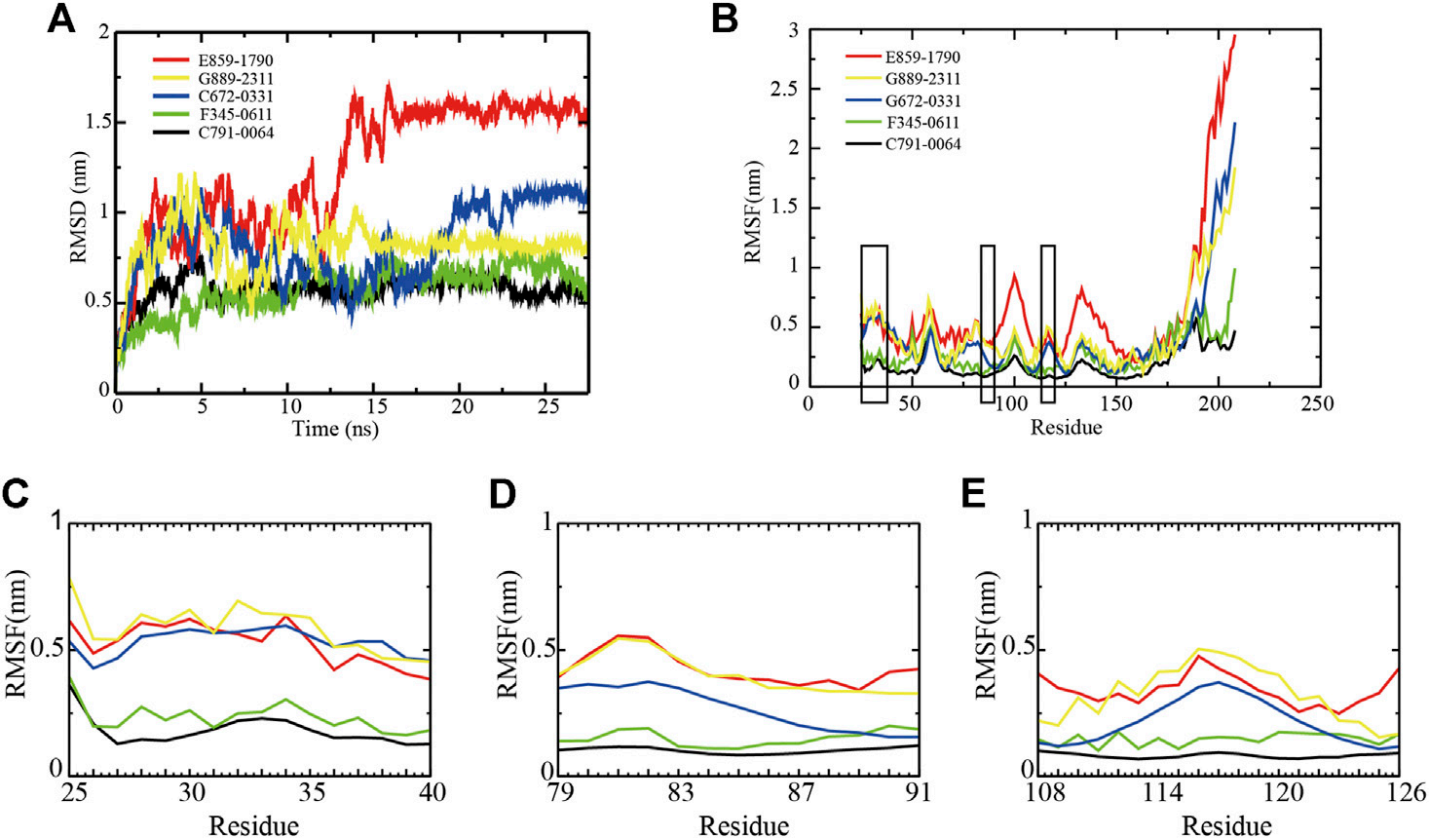
Backbone RMSD and RMSF values of RAD52–ligand complexes. **(A)** RMSD of the five RAD52–ligand complexes during a 27.5 ns simulation. **(B)** RMSFs of the amino acids within the RAD52 self-association domain during three equal portions of a 27.5 ns molecular dynamics simulation of RAD52–ligand complexes. **(C–E)** presented the enlarged fluctuation of three segments (Cys25-Gln40, Phe79-Gln91, and Cys108-Tyr126) containing the key amino acids in the RAD52 self-association binding sites involved in RAD52–ligand interaction.

The backbone root-mean-square deviation (RMSD) value indicates the stability of RAD52–ligand complexes. As shown in Figure 2A, although E859-1790, C672-0331, and G889-2311 systems were able to reach equilibrium after 16 ns, 23 ns, and 15 ns, respectively, the fluctuations were wider at the beginning of the simulation, compared with F345-0611 and C791-0064. The difference between the peaks and troughs of the RMSD value of the three systems was 1.2 nm, 0.77 nm, and 0.79 nm, respectively, suggesting unstable binding of the compounds to RAD52. F345-0611 and C791-0064 systems reached a stable state after 5 ns (Figure 2A). The difference between the peaks and troughs was 0.56 nm and 0.30 nm, respectively, and the mean of RMSD values were 0.60 nm and 0.58 nm, respectively. The results of the RMSD analysis suggested that the C791-0064 system was the most stable one among the five systems, followed by F345-0611.

The root-mean-square fluctuation (RMSF) value demonstrates the flexibilities of the RAD52–ligand complexes (Figure 2B). A lower RMSF value indicates a higher stability of the amino acids being analyzed. As shown in Figure 2B, the fluctuations of amino acid residues 25–175 in the five complexes were relatively consistent, peaking at around residues Gln30, Gly59, Asn100, and Ala133. We then focused on the RMSF values of the key amino acids that contributed to the RAD52–compound interaction as identified by the LigPlot + analysis (Supplementary Figure S2). The enlarged fluctuations of three segments containing their residues are presented in Figures 2C–E. Among the five systems, the C791-0064 system had the lowest fluctuations in all of the three segments analyzed. Except for Phe26, the other key self-association amino acid residues in the C791-0064 system had the lowest fluctuations (Supplementary Table S3), indicating the highest stability of the RAD52 self-association domain during molecular dynamics simulation upon the binding of C791-0064.

The total energies of the RAD52–ligand complexes were then calculated according to the MD trajectory file (Supplementary Figure S3). Among the five complexes analyzed, RAD52-C791-0064 gave the lowest total energy value, suggesting that C791-0064 binding to RAD52 is the most stable one among the five putative inhibitors.

### BRCA2-Deficient Cell Line Was More Sensitive to C791-0064 Treatment

**FIGURE 3:**
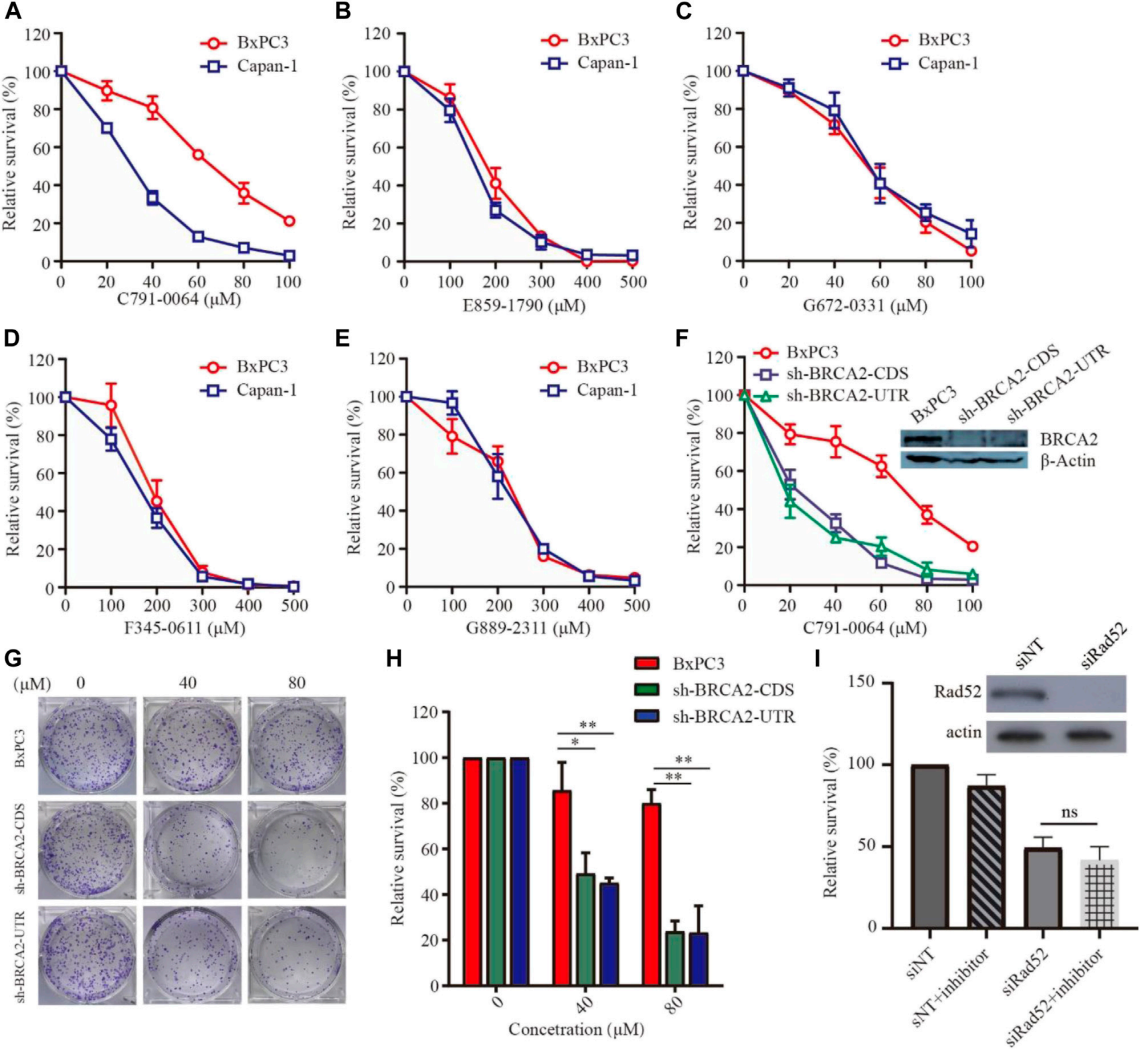
Effect of the putative RAD52 inhibitors on the survival of BRCA2 wild-type and deficient cells. Capan-1(BRCA2-deficient) and BxPC3 (BRCA2 wild type) cells were treated with different concentrations of the five compounds for 72 h, **(A)** C791-0064, **(B)** E859-1790, **(C)** G672-0331, **(D)** F345-0611, and **(E)** G889-2311 **(F)** (*n* = 3). C791-0064 treatment of BRCA2-knockdown BxPC3 cells (sh-BRCA2-CDS and sh-BRCA2-UTR) and wild-type BxPC3 cells for 72 h (*n* = 3). CCK8 assay was performed to evaluate the cell survival. **(G,H)** Clonogenic assay performed with BRCA2 wild-type or BRCA2-knockdown cells and its quantification (*n* = 3). Error bars represent SDs. **(I)** siRNA knockdown of RAD52 in sh-BRCA2-CDS cells and treatment with C791-0064.

To evaluate the efficacy of the newly identified putative RAD52 inhibitors, we first treated patient-derived BRCA2-proficient (BxPC3) and BRCA2-deficient (Capan-1) pancreatic cancer cells (Figure 3). C791-0064 displayed a differential inhibitory effect on the proliferation of BxPC3 and Capan-1 cells, but not the other four compounds tested. The IC50 of C791-0064 on BxPC3 cells (64.62 μM) was approximately 2 times higher than that for Capan-1 cells (28.92 μM). At 60 μM, Capan-1 proliferation was almost completely inhibited by C791-0064, while more than 50% of BxPC3 cells survived. (Figure 3A). Other four compounds (E859-1790, F345-0611, G672-0331, and G889-2311) had no differential inhibitory effect on the proliferation of BxPC3 and Capan-1 cells (Figures 3B–E). Because BxPC3 and Capan-1 cells have different genetic backgrounds, which might result in a differential response to C791-0064 treatment, we established BRCA2 knockdown–stable cell lines by lentiviral transduction to confirm the result shown in Figure 3A. Treatment of BRCA2-knockdown cells with C791-0064 revealed a significant decrease in cell viability, compared with the wild-type cells (Figure 3F). Plate colony formation assay showed that C791-0064 inhibited the colony formation of BRCA2 knockdown cells significantly, compared with BRCA2 wild-type BxPC3 cells, indicating that C791-0064 can inhibit BRCA2-deficient cells (Figures 3G,H). To analyzed whether C791-0064 was specifically acting on RAD52, we transfected BRCA2-deficient cells (sh-BRCA2-CDS) with either nontargeting siRNA or siRAD52 and then treated them with C791-0064. As shown in Figure 3I, siRAD52 transfection significantly inhibited the survival of BRCA2-deficient cells, compared with the cells transfected with nontargeting siRNA. The treatment with 40 μM of C791-0064 did not further reduce the survival of the cells, suggesting the specific inhibition of the compound to RAD52.

### C791-0064 Specifically Inhibited RAD52 Protein Self-Association *In Vitro*

**FIGURE 4:**
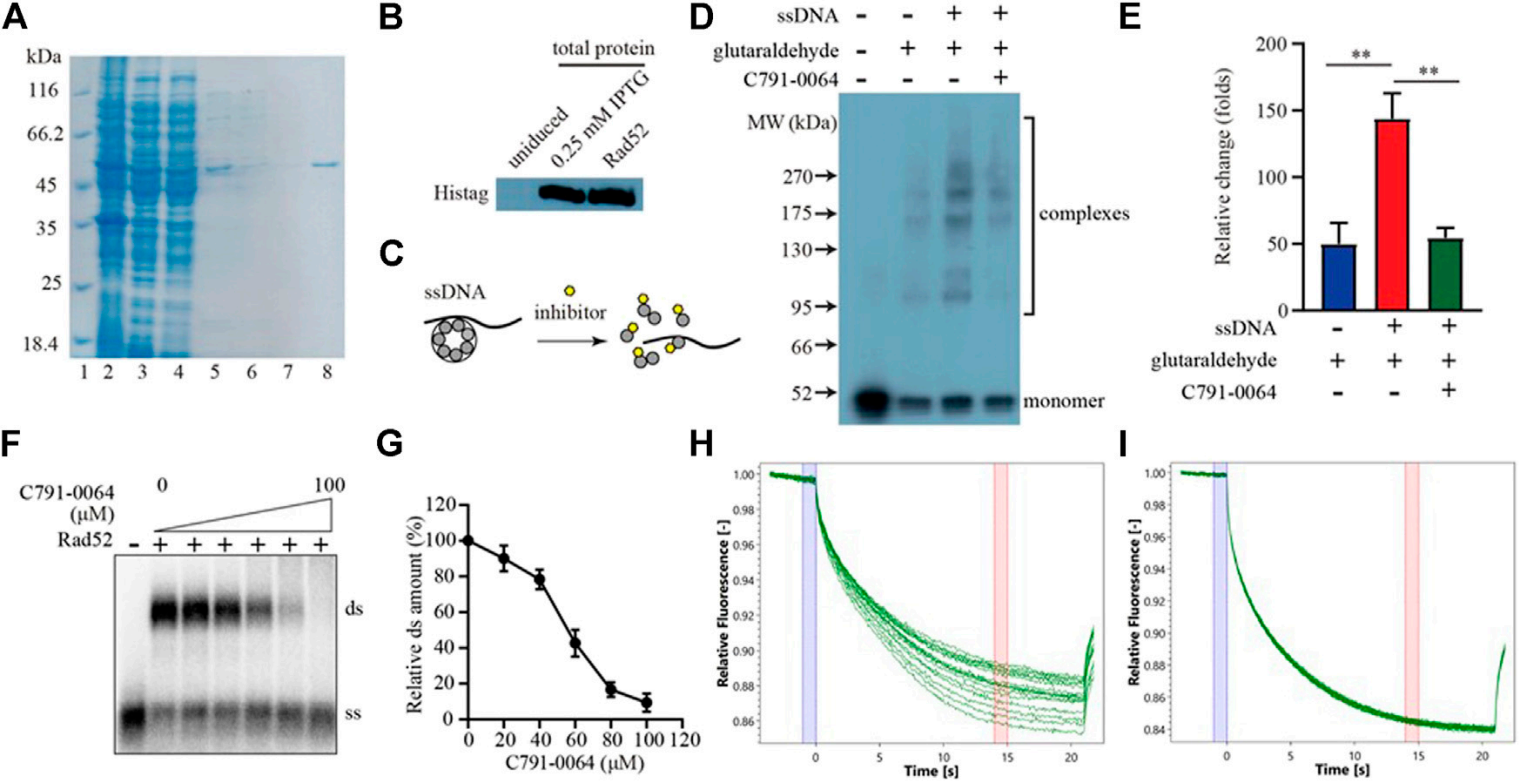
C791-0064 inhibited RAD52 self-association. **(A)** Purification of human RAD52 protein analyzed by SDS-PAGE. Lane 2, total cell lysate; lane 3, supernatant; lane 4, Ni sepharose flow-through; lane 5, Ni sepharose eluate; lane 6, heparin flow-through; lane 7, heparin wash; and lane 8, heparin eluate. **(B)** Western blot analysis of RAD52 expressing plasmid-transformed *E. coli* cell lysate and purified RAD52 protein with anti-Histag antibody. **(C)** Schematic illustration of the electrophoretic mobility shift assay (EMSA). **(D)** Western blot of the EMSA result detected with anti-Histag antibody. **(E)** Quantification of the relative amount of polymer protein; the values were normalized by setting the value of no C791-0064, glutaraldehyde, and ssDNA reaction to be 100% (*n* = 3, error bars stands for SD). **(F,G)** Single-strand annealing efficiency of RAD52 in the presence of C791-0064. ds, double strand; ss, single strand. **(H,I)** Microscale thermophoresis (MST) analysis of C791-0064 **(H)** and G672-0031 **(I)** binding to Rad52 protein.

RAD52 needs to form a heptamer to be functional *in vivo*. The docking site we chose for virtual screening was predicted to be critical for RAD52 heptamer formation. We, therefore, analyzed the impact of C791-0064 on RAD52 self-association *in vitro* to determine whether the synthetic lethality observed was indeed resulted from the disruption of RAD52 function. Human RAD52 protein with 6-histidine affinity tag was expressed in *E. coli* and purified to homogeneity, as shown in Figures 4A,B. We then exploited an electrophoretic mobility shift assay (EMSA) to investigate whether C791-0064 had an effect on RAD52 self-association (Figure 4C). Without ssDNA stimulation, the majority of RAD52 was in the form of a monomer (Figure 4D, lane 2). When ssDNAs were added to the system, a large amount of RAD52 was present in a higher molecular weight state (Figure 4D, lane 3). Under the same condition, the addition of C791-0064 inhibited RAD52 complex formation (Figure 4D, lane 4). We also analyzed the effect of the other four compounds which did not show any differential inhibition on the BRCA2-deficient cells (Supplementary Figure S4). Compared to C791-0064, the inhibition of RAD52 complex formation by the other four compounds were not as efficient as C791-0064. Therefore, C791-0064 inhibited the formation of higher molecular weight RAD52 complexes, which is a possible explanation to its differential inhibitory effect on the BRCA2 wild-type and loss-of-function cells. Then, we performed single-strand annealing experiments to see whether the activity of RAD52 was impaired by C791-0064. As shown in Figures 4F,G, C791-0064 inhibited the formation of double-stranded (ds) product, which was a result of RAD52 single-strand annealing activity. Further, microscale thermophoresis (MST) analysis indicated a direct binding of C791-0064° RAD52 (Figure 4H), which was not the case for G672-0031 (Figure 4I).

### C791-0064 Induced Deoxyribonucleic Acid Damage and Apoptosis More Severely in BRCA2-Deficient Cells

**FIGURE 5:**
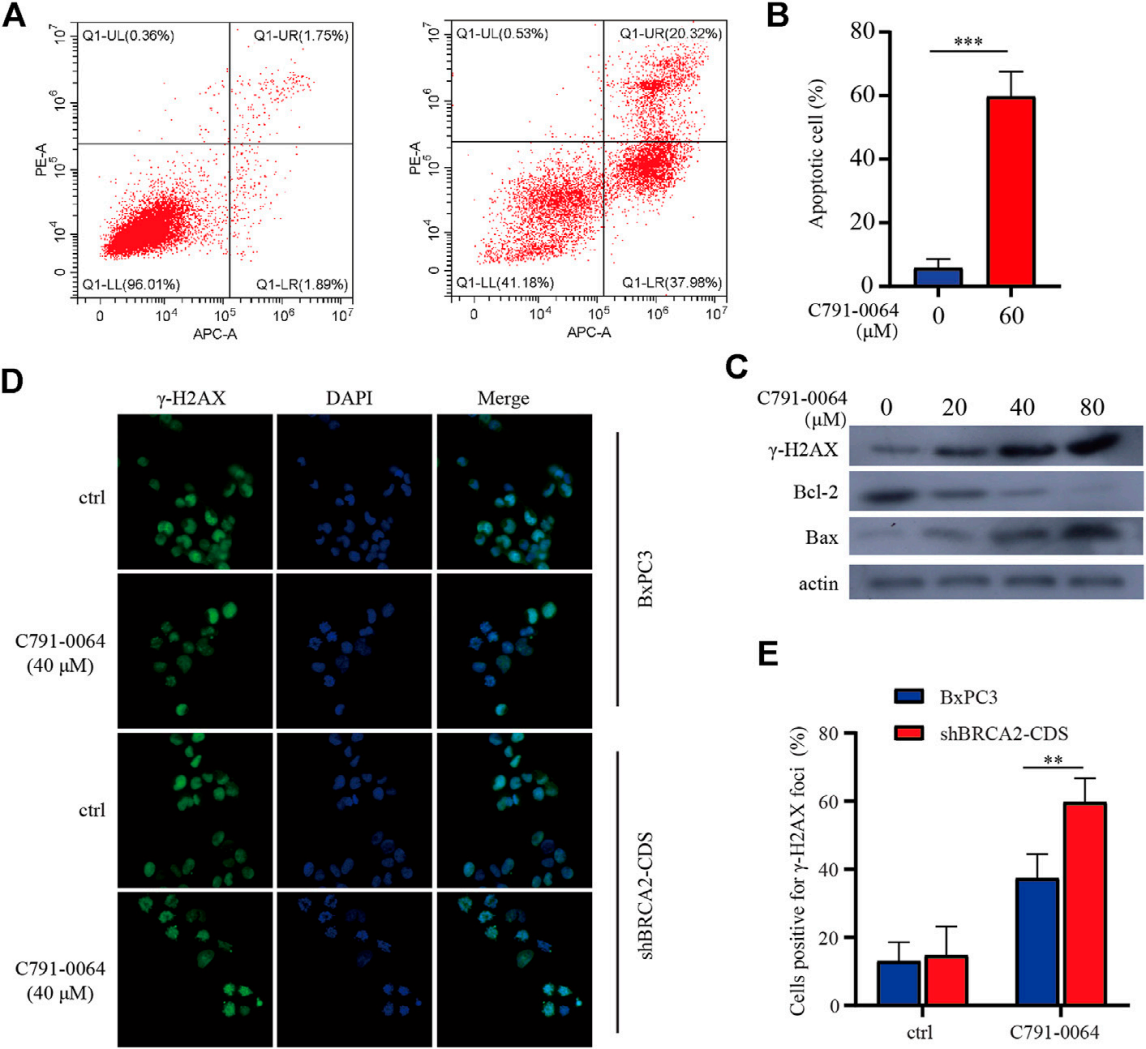
C791-0064 caused apoptosis of BRCA2-deficient cells. **(A)** Flow cytometry detection of apoptosis after 40 μM of C791-0064 treatment of shBRCA2-CDS BxPC3 cells for 48 h. **(B)** Quantification of apoptotic cell percentage (*n* = 3). **(C)** Western blot analysis of shBRCA2-CDS BxPC3 cells treated with different concentrations of C791-0064 for 48 h. **(D,E)** Immunofluorescence staining of γ-H2AX in shBRCA2-CDS BxPC3 and BxPC3 cells treated with 40 μM of C791-0064 for 24 h and its quantification for cells positive forγ-H2AX foci (*n* = 3). Error bars represent for SD.

Treatment of BRCA2 knockdown cells with C791-0064 revealed that the apoptotic cell percentage increased significantly upon 40 μM of C791-0064 (Figures 5A,B). Next, we determined the expression level of apoptotic markers upon C791-0064 treatment. As shown in Figure 5C, as the concentration C791-0064 increased, Bax expression was upregulated, whereas Bcl-2 expression was downregulated, indicating an increased apoptosis in these cells. γH2AX is a DNA double-stranded damage marker protein; the accumulation of γH2AX was observed in BRCA2-deficient cells treated with C791-0064, indicating that C791-0064 caused DNA double-strand break accumulation in these cells (Figure 5C). This result was further supported by our immunofluorescence assay. C791-0064–treated BRCA2-deficient cells had significantly increased γH2AX foci, compared with the untreated or BRCA2 wild-type cells (Figures 5D,E). Based on these results, it is likely that the inhibition of the RAD52 function by C791-0064 in BRCA2-deficient cells leads to inefficient repair of double-strand breaks and induces apoptosis.

## Discussion

Even though the function of higher eukaryotes RAD52 in homologous recombination and other DNA repair pathways is getting clearer with progresses in genetics and biochemical studies (Benson et al., 1998; Mcilwraith and West, 2008; Jalan et al., 2019), its exact role in BRCA-deficient mammalian cells has not been fully dissected. Different from BRCA2, RAD52 monomers form a ring-shaped oligomer, mostly heptamer, while carrying out its function in homologous recombination (Stasiak et al., 2000; Shinohara et al., 2010). Several studies have demonstrated that RAD52 is required for the survival of cells with loss-of-function mutation in genes such as BRCA1/2, PALB2, and RAD51 paralogs, which is not the case for normal cells. This differential effect renders RAD52 as a potential therapeutic target to treat cancers with such mutations (Feng et al., 2011; Chun et al., 2013).

In the current study, we identified and characterized a RAD52-specific small-molecule inhibitor, C791-0064, from 66,608 compounds by computer-aided virtual screening, molecular dynamics simulation, and biochemical assays. Compared to BRCA2-proficient BxPC3 cells, C791-0064 inhibited the proliferation of patient-derived BRCA2-deficient Capan-1 cells and shRNA-mediated BRCA2 knockdown BxPC3 cells more significantly, supporting the synthetic lethality between RAD52 inhibition and BRCA2 loss of function. We have demonstrated through *in vitro* experiments that C791-0064 can inhibit the formation of the RAD52 complex, which is believed to be essential for RAD52 to execute its physiological function in DNA repair (Kwon and Sung, 2017). C791-0064 was shown to induce apoptosis by causing the accumulation of DNA double-strand breaks.

Recently, small-molecule inhibitors targeting RAD52 have been exploited using different approaches. Huang et al. (2016) performed fluorescence-based high throughput screening for 372,903 compounds and identified a series of candidate RAD52 inhibitors for further development. Hengel et al. (2016) exploited *in silico* screening to discover RAD52 inhibitors that disrupt RAD52–ssDNA interaction. Sullivan et al. (2016) screened 140,952 FDA-approved drugs and drug-like compounds with virtual screening to find new RAD52 inhibitors. These three studies focused on the ssDNA binding activity of RAD52. Different from their work, we chose a different docking site, which is critical to the formation of the RAD52 complex according to the result from biochemical and structural biology studies. Also, we focused on different small-molecule libraries, enabling the identification of new chemical compounds. Prior to the current study, there was another work by Chandramouly et al. (2015), which identified 6-OH-dopa as a specific inhibitor to RAD52 ring structure formation. Importantly, 6-OH-dopa was found to disrupt the undecamer ring of truncated RAD52 (residues 1–209) into dimers, suggesting a unique conformation of the RAD52 ring structure that has not yet been fully understood in relation to its function in DNA repair.

In our previous study, we screened 47,737 compounds from the Chemdiv Targeted Diversity Library (TDL) to discover inhibitors targeting the ssDNA binding and DNA strand exchange activities of RAD52 (Jian et al., 2018). We showed that F779-0434 selectively inhibited the proliferation of BRCA2-deficient cells, and an *in vitro* pull-down assay demonstrated that F779-0434 efficiently disrupted the association of ssDNA with RAD52. This shows that our virtual screening method is stable and reliable.

Our current research on C791-0064 is still *in vitro*, and the concentration required for efficient inhibition of BRCA2-deficient cells was still relatively high. Structural modification of C791-0064 may be a good strategy to reduce the concentration of action. Since the small-molecule inhibitors are prone to be off-target, it is necessary to determine the specificity of C791-0064 by analyzing whether it binds to RAD52 homologous protein or other structurally similar proteins to some extent. In conclusion, C791-0064 identified in the current study could serve as a promising leading compound and worthy to be further exploited for the development targeted therapy for BRCA2-deficient tumors.

## Data Availability

The original contributions presented in the study are included in the article/Supplementary Material, further inquiries can be directed to the corresponding authors.
