# Supplementary figures and images for: Identification of a RAD52 Inhibitor Inducing Synthetic Lethality in BRCA2-Deficient Cancer Cells
Source: Front Pharmacol. 2021 Apr 29;12:637825. doi: 10.3389/fphar.2021.637825 (PMC8118686; doi:10.3389/fphar.2021.637825)

**Table S1. Structures of top 28 chemicals.**

| 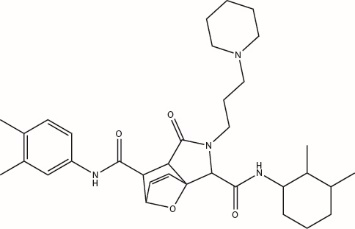 | 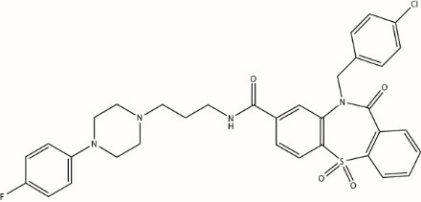 | 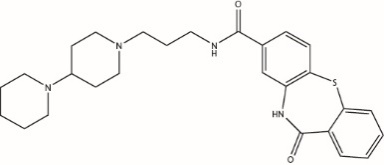 |
| --- | --- | --- |
| C073-3433 | C530-1040 | C791-0064 |
| 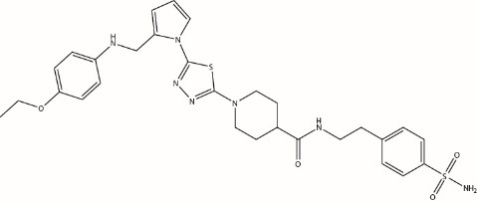 | 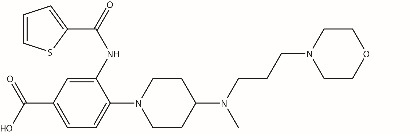 | 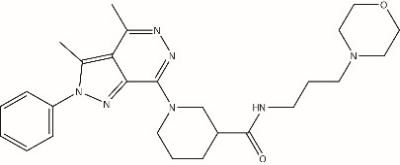 |
| C794-1601 | F085-0524 | E859-1790 |
| 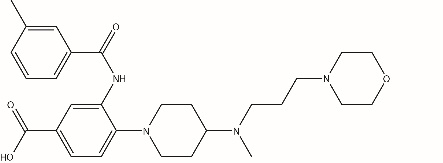 | 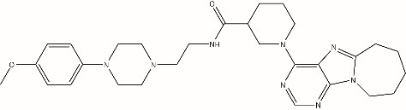 | 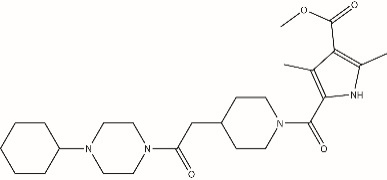 |
| F085-0454 | E715-0077 | F862-0179 |
| 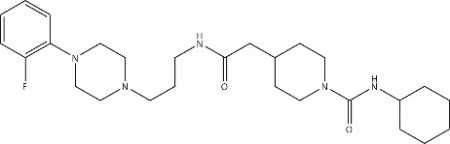 | 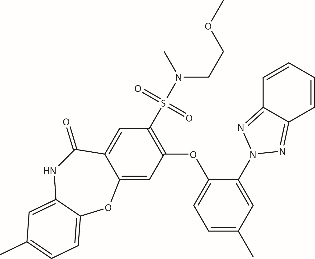 | 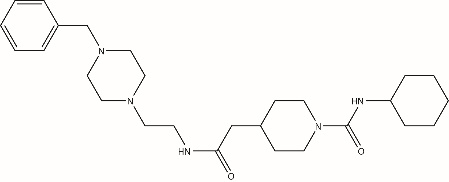 |
| F345-0586 | J030-1084 | F345-0611 |
| 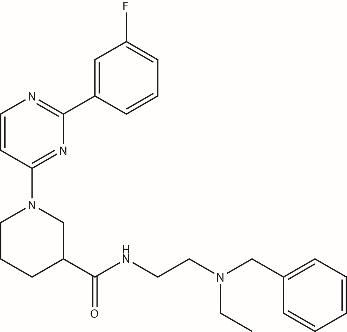 | 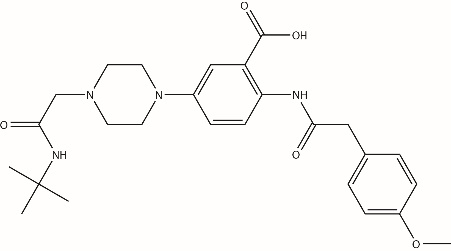 | 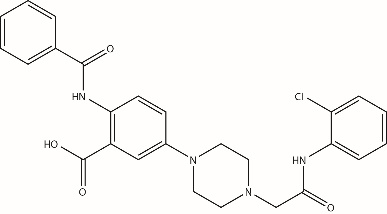 |
| F726-1008 | F687-1117 | F687-0800 |
| 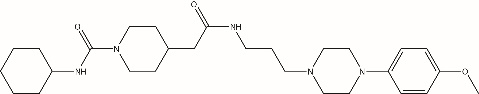 | 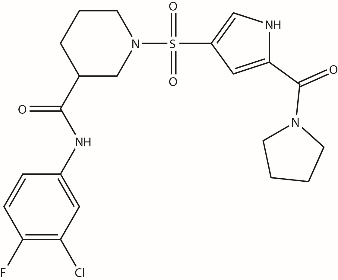 | 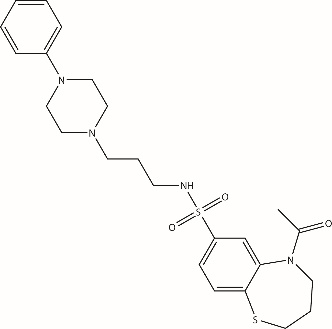 |
| F345-0581 | F899-0160 | G269-0315 |
| 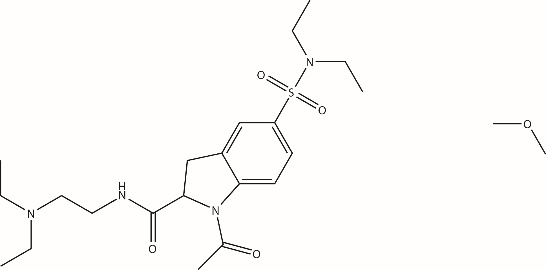 | 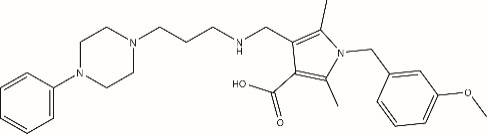 | 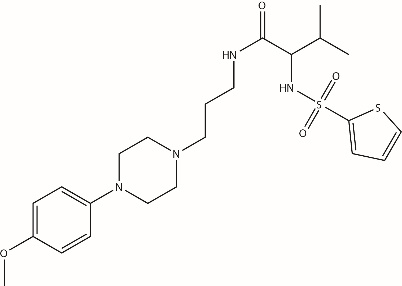 |
| G384-0676 | G396-1121 | G557-0245 |
| 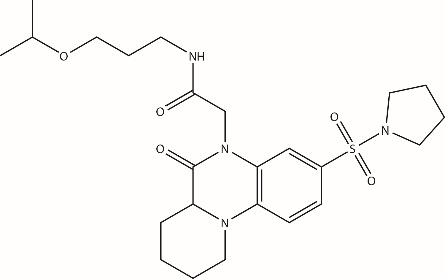 | 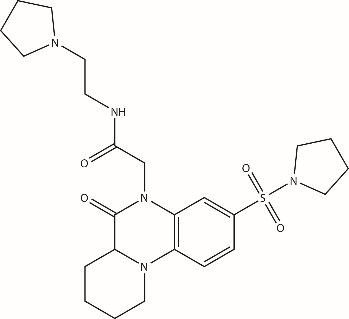 | 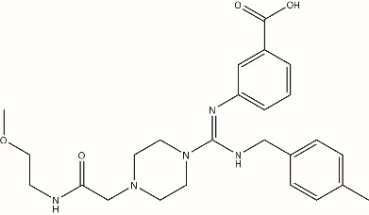 |
| G672-0319 | G672-0331 | G696-5877 |
| 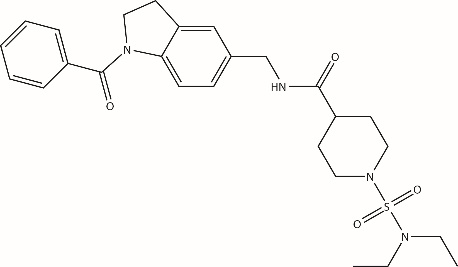 | 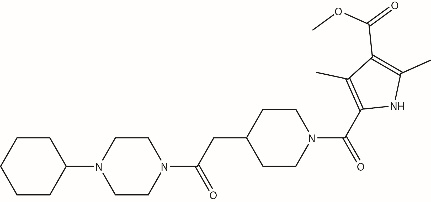 | 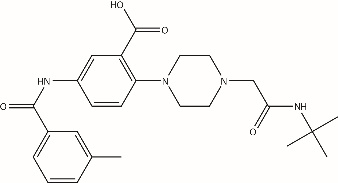 |
| G883-0652 | G883-1540 | G889-2311 |
| 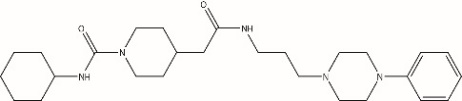 |  |  |
| F345-0600 |  |  |

Supplement: Supplementary file 1 [file table1.docx]
