# Supplementary material for: Identification of a RAD52 Inhibitor Inducing Synthetic Lethality in BRCA2-Deficient Cancer Cells
Source: Front Pharmacol. 2021 Apr 29;12:637825. doi: 10.3389/fphar.2021.637825 (PMC8118686; doi:10.3389/fphar.2021.637825)
Supplement: Supplementary file 2 [file table2.docx]

**Table S2. ADMET properties of top 28 chemicals in docking analysis.**

| Class | Name | Docking Score | Water Solubility | Molecular weight | Consensus Log Po/w | TPSA | Log Kp (skin permeation) | Bioavailability Score | Synthetic accessibility |
| --- | --- | --- | --- | --- | --- | --- | --- | --- | --- |
| 1 | C791-0064 | -38.3559 | Moderately soluble | 478.65 | 0.65 | 89.98 Å² | -6.37 cm/s | 0.55 | 4.57 |
|  | J030-1084 | -34.2799 | Poorly soluble | 599.67 | 4.19 | 133.26Å² | -6.39 cm/s | 0.55 | 4.55 |
|  | G269-0315 | -37.6489 | Moderately soluble | 488.67 | 1.74 | 106.64 Å² | -7.24 cm/s | 0.55 | 4.26 |
|  | C530-1040 | -37.3533 | Poorly soluble | 647.16 | 3.91 | 98.41 Å² | -6.48 cm/s | 0.55 | 4.62 |
|  | E715-0077 | -36.0349 | Moderately soluble | 532.68 | 1.61 | 91.65 Å² | -7.45 cm/s | 0.55 | 4.76 |
| 2 | C073-3433 | -36.696 | Moderately soluble | 576.77 | 2.91 | 90.98 Å² | -6.91 cm/s | 0.55 | 7.26 |
| 3 | C794-1601 | -36.1206 | Moderately soluble | 609.76 | 3.24 | 181.09 Å² | -7.39 cm/s | 0.55 | 4.85 |
| 4 | E859-1790 | -35.5769 | Soluble | 477.6 | 1.69 | 88.41 Å² | -7.65 cm/s | 0.55 | 4.29 |
| 5 | F085-0524 | -40.9443 | Soluble | 486.63 | -0.73 | 113.59 Å² | -8.74 cm/s | 0.55 | 4.11 |
|  | F085-0454 | -35.4757 | Soluble | 494.63 | -0.48 | 85.35 Å² | -8.54 cm/s | 0.55 | 4.11 |
|  | F687-1117 | -34.7175 | Soluble | 482.57 | 1.05 | 111.21 Å² | -8.73 cm/s | 0.55 | 3.4 |
|  | F687-0800 | -34.7161 | Soluble | 492.95 | 1.81 | 101.98 Å² | -7.87 cm/s | 0.55 | 3.13 |
|  | G696-5877 | -36.1025 | very soluble | 467.56 | 0.39 | 106.50 Å² | -9.51 cm/s | 0.55 | 3.89 |
|  | G889-2311 | -35.8642 | Soluble | 452.55 | 1.16 | 101.98 Å² | -8.61 cm/s | 0.55 | 3.19 |
|  | G396-1121 | -35.4963 | Soluble | 490.64 | 0.13 | 69.97 Å² | -8.14 cm/s | 0.55 | 3.7 |
| 6 | F345-0611 | -39.9137 | Soluble | 469.66 | -0.65 | 67.92 Å² | -7.25 cm/s | 0.55 | 3.38 |
|  | F345-0600 | -37.3766 | Moderately soluble | 469.66 | 2.01 | 67.92 Å² | -6.79 cm/s | 0.55 | 3.55 |
|  | F345-0581 | -36.2842 | Moderately soluble | 499.69 | 2.05 | 77.15 Å² | -7.00 cm/s | 0.55 | 3.71 |
|  | F345-0586 | -35.2196 | Moderately soluble | 487.65 | 2.34 | 67.92 Å² | -6.83 cm/s | 0.55 | 3.68 |
| 7 | F899-0160 | -36.52 | Moderately soluble | 482.96 | 2.56 | 110.96 Å² | -7.68 cm/s | 0.55 | 3.84 |
| 8 | F726-1008 | -36.1218 | Moderately soluble | 461.57 | 2.87 | 61.36 Å² | -6.21 cm/s | 0.55 | 3.82 |
| 9 | F862-0179 | -34.7145 | Moderately soluble | 472.63 | 1.89 | 85.95 Å² | -7.11 cm/s | 0.55 | 4.45 |
| 10 | G883-1540 | -39.4184 | Moderately soluble | 472.62 | 3.14 | 85.95 Å² | -7.11 cm/s | 0.55 | 4.45 |
|  | G883-0652 | -35.9282 | Moderately soluble | 498.64 | 2.81 | 98.41 Å² | -7.59 cm/s | 0.55 | 3.51 |
|  | G384-0676 | -38.9443 | Soluble | 438.58 | 0.9 | 98.41 Å² | -8.02 cm/s | 0.55 | 3.94 |
| 11 | G672-0319 | -38.4009 | Soluble | 492.63 | 2 | 107.64 Å² | -7.83 cm/s | 0.55 | 4.39 |
|  | G672-0331 | -35.1261 | Soluble | 489.63 | 0.65 | 101.65 Å² | -8.15 cm/s | 0.55 | 4.37 |
| 12 | G557-0245 | -37.9544 | Moderately soluble | 494.67 | 3.14 | 127.60 Å² | -6.78 cm/s | 0.55 | 4.35 |
