# Supplementary material for: Identification of a RAD52 Inhibitor Inducing Synthetic Lethality in BRCA2-Deficient Cancer Cells
Source: Front Pharmacol. 2021 Apr 29;12:637825. doi: 10.3389/fphar.2021.637825 (PMC8118686; doi:10.3389/fphar.2021.637825)
Supplement: Supplementary file 3 [file datasheet1.pdf]

Figure S1

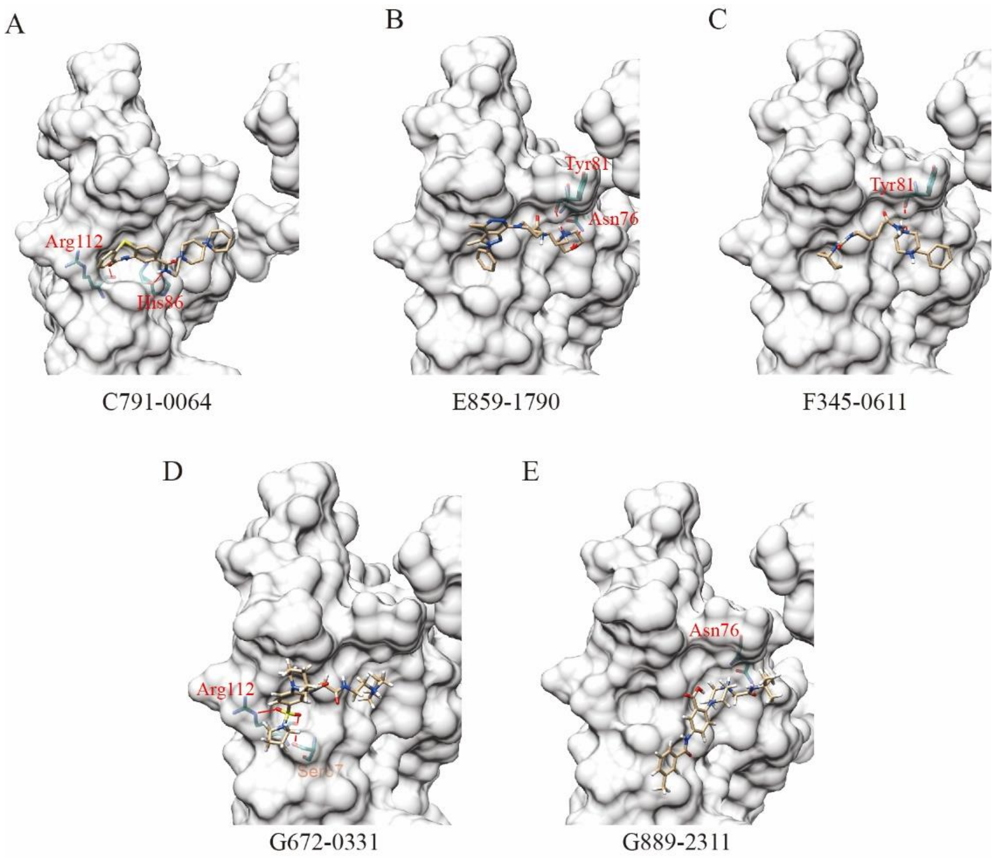

Figure S1. RAD52–ligands complexes structures. ((A) C791-0064, (B) E859-1790, (C) F345-0611, (D).G672-0331 and (E) G889-2311). The ligands and amino acid residues of RAD52 involved in hydrogen bonding are presented as stick models. The dashed red lines represent the hydrogen bonds predicted by the LigPlot+ software.

Figure S2

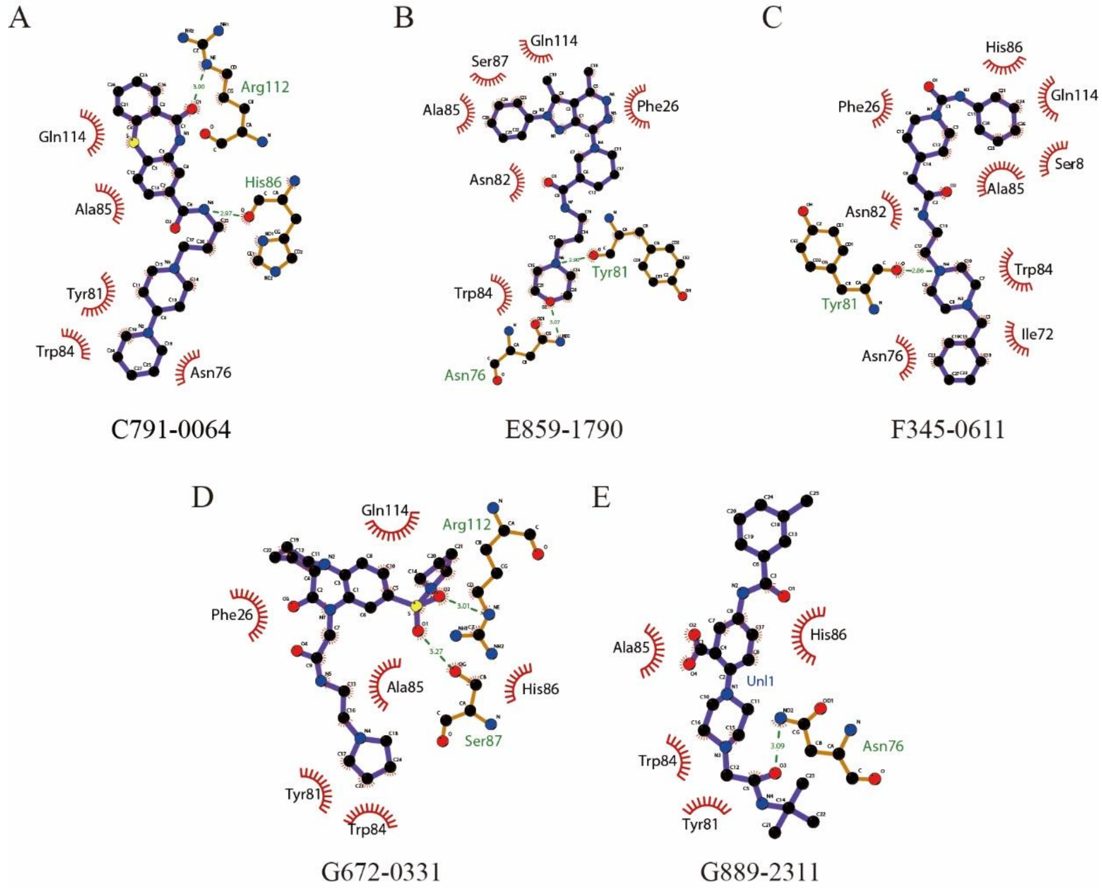

Figure S2. 2D diagrams of the interactions between RAD52 and the five candidate inhibitors. Hydrophobic interactions are presented by red arcs. Hydrogen bonds are indicated by dashed green lines. Ligands are presented in purple. C, N, O, and S atoms are shown in black, blue, red, and yellow, respectively. The graphs were generated using the LigPlot+ software.

Figure S3

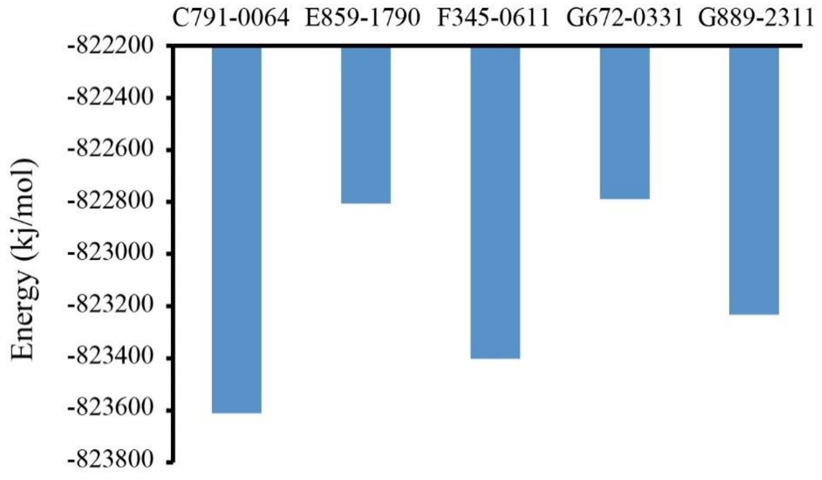

Figure S3. Comparison of the binding free energies values of the RAD52-ligands association.

Figure S4

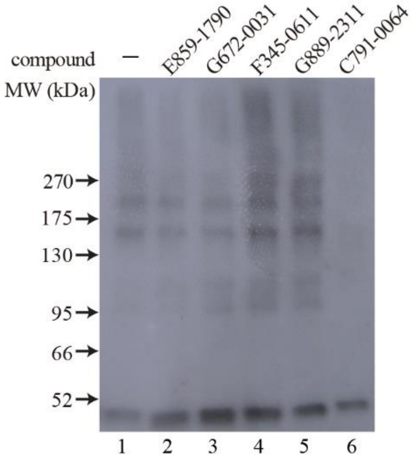

Figure S4. Comparison of the inhibition on RAD52 complex formation by small molecule compounds. The EMSA assay was performed as described in the Material and Methods. C791-0064 or the other four small molecule compounds was added to the reaction to investigated their inhibitory effect on RAD52 complex formation.
