# Supplementary material for: Identification of a RAD52 Inhibitor Inducing Synthetic Lethality in BRCA2-Deficient Cancer Cells
Source: Front Pharmacol. 2021 Apr 29;12:637825. doi: 10.3389/fphar.2021.637825 (PMC8118686; doi:10.3389/fphar.2021.637825)
Supplement: Supplementary file 4 [file table3.docx]

**Table S3. The RMSF value of self-association binding related key amino acid residues**

| key AA residues | C859-1790 | C672-0311 | C791-0064 | G889-2311 | F345-0611 |
| --- | --- | --- | --- | --- | --- |
| PHE26 | 0.4873 | 0.4278 | 0.2079 | 0.5434 | 0.1977 |
| ILE72 | 0.4254 | 0.2609 | 0.1090 | 0.2812 | 0.1751 |
| ASN76 | 0.4817 | 0.3329 | 0.1008 | 0.4096 | 0.1811 |
| TYR81 | 0.5563 | 0.3540 | 0.1167 | 0.5456 | 0.1855 |
| ASN82 | 0.5487 | 0.3745 | 0.1154 | 0.5341 | 0.1904 |
| TRP84 | 0.3998 | 0.3086 | 0.0891 | 0.3971 | 0.1114 |
| ALA85 | 0.3866 | 0.2738 | 0.0847 | 0.4001 | 0.1103 |
| HIS86 | 0.3820 | 0.2374 | 0.0870 | 0.3503 | 0.1298 |
| SER87 | 0.3600 | 0.2020 | 0.0914 | 0.3503 | 0.1301 |
| ARG112 | 0.3266 | 0.1836 | 0.0735 | 0.3763 | 0.1756 |
| GLN114 | 0.3556 | 0.2618 | 0.0729 | 0.4138 | 0.1508 |
